# Hospital-mandated restrictions during the COVID-19 pandemic alter hospital-acquired viral respiratory infections during hematopoietic stem cell transplantation: a single center pediatric experience

**DOI:** 10.1017/ash.2025.10240

**Published:** 2025-12-01

**Authors:** Perry Morocco, Madan Kumar, Miranda Foote, Caitlin Cohen, Michelle Nassin, John Cunningham, James LaBelle

**Affiliations:** 1 Department of Pediatrics, Section of Hematology/Oncology/Stem Cell Transplantation, University of Chicago, Chicago, IL, USA; 2 Department of Infectious Disease, Comer Children’s Hospital, University of Chicago, Chicago, IL, USA

## Abstract

**Objective:**

Evaluating the COVID-19 pandemic restrictions and their effect on rates of new respiratory viral infections in patients undergoing hematopoietic stem cell transplantation (HSCT), and if there were changes to the morbidity and mortality rates compared to historical controls.

**Design:**

Retrospective chart review.

**Setting:**

University-based tertiary care center.

**Patients:**

All patients who underwent HSCT, including chimeric antigen receptor T-cell therapy, between 7/1/2013 and 12/19/2021 were included in the study. No eligible patients were excluded.

**Results:**

We found a significant difference in new respiratory infections as measured by a respiratory viral pathogen (RVP) polymerase chain reaction-based assay during transplant admissions between the pre- and early COVID-19 eras, with complete absence of new respiratory viral infections in the early COVID-19 era. The late-COVID-19 era, coincident with availability of severe acute respiratory syndrome coronavirus 2 vaccines and relaxation of some hospital-based restrictions, had a similar incidence of new RVP positivity compared to the pre-COVID-19 era. For allogeneic HSCT outcomes alone, rates of pediatric intensive care unit admission (66.7% vs 32.2%, *p* = .01), intubation (57.1% and 18.6%, *p* < .01), and oxygen requirement (66.7% vs 40.7%, *p* = .04) were found to be statistically different with new RVP positivity. Other outcomes examined, including death at 100/180/365 days post-transplant, length of stay, and acute graft-versus-host disease incidence, were similar, regardless of new RVP positivity.

**Conclusions:**

In order to reduce morbidity, these findings argue for a continuation of the strict protective isolation practices initially employed during the pandemic for HSCT patients, particularly during viral emergence following the COVID-19 lockdown.

## Introduction

With the COVID-19 pandemic, healthcare systems across the world instituted large systemwide changes to protect patients and staff from the novel coronavirus, severe acute respiratory syndrome coronavirus 2 (SARS-CoV-2).^1,2^ The World Health Organization ultimately identified SARS-CoV-2 as a public health emergency worldwide on February 11, 2020,^3^ and initially recommended preventative measures such as social distancing, avoiding unnecessary travel, and regular hand washing.^4^ The US Centers for Disease Control and Prevention soon recommended more stringent precautions, including social distancing combined with masks covering the nose and mouth for people over age 2 years old and regular hand washing to aid in reducing the spread of COVID-19.^5^ The American Society for Transplantation and Cellular Therapy issued further recommendations for universal testing of all admitted patients, daily screening of healthcare workers, dedicated COVID-19 units, negative pressure rooms for positive patients, and limiting of hospital visitors for bone marrow transplant units.^6^ Combined with universal precautions, the emphasis was on preventing transmission of this highly contagious pathogen.^6^

Although the importance of hand hygiene is well known, data has been emerging on the prevention and rates of non-COVID-19 nosocomial infections following the start of the COVID-19 pandemic.^7,8^ Aggressive infection control strategies can reduce the rates of respiratory viral infections in immunocompromised patients,^9^ including universal masking in preventing hospital-acquired viral infections in hematopoietic stem cell transplantation (HSCT) units.^10,11^ As the pandemic progressed, more was understood about how stem cell transplanted patients are affected by SARS-CoV-2, beyond a clear increased risk of mortality.^12^ However, data regarding pediatric transplanted patients has been limited.^13^ A case series of the Spanish Group of Transplant found that children are less likely to be infected by SARS-CoV-2 but still suffer from high rates of mortality.^13^ One institution discovered that in their adult allogeneic HSCT patients, there was a significant decrease in seasonal respiratory viral infections during the pandemic.^8^ Combined, these findings highlight the benefits of stricter infection control practices and expanded PPE use for HSCT patients, such as visitor restrictions and limitations of hospital staff entering rooms. As pediatric patients are more likely to acquire respiratory viral infections at baseline while inpatient, and respiratory viral infections confer significant morbidity and mortality such as delayed time to engraftment,^14,15^ which is exacerbated if infections progress to the lower respiratory tract,^16^ further knowledge gains on how to prevent such seasonal infections are critical to improve outcomes. Thus, identifying new practice changes to minimize the risk of nosocomial infections could lead to a reduction in infections in pediatric hematopoietic stem cell transplant patients, particularly following the emergence of SARS-CoV-2 and disruption of the normal URI seasonality patterns.^17^

Infection control measures, including proper hand hygiene, patient isolation if positive for a viral respiratory infection, and general neutropenic precautions, are critical in preventing infections in immunocompromised patients.^18–20^ Reports have emerged on how increased infection control measures, such as universal masking, visitor restrictions and screening, and increased awareness from the pandemic altered rates of nosocomial infections, including 1 institution that measured a 19.6% decrease in their total nosocomial infection rate during the pandemic.^7^

Testing also changed during the pandemic, with rapid development of nucleic acid and antigen-based tests.^21^ Additionally, retrospective evidence emerged that non-SARS-CoV-2 viral infections decreased during this period for both inpatients and patients in the community,^8,22,23^ likely due to an increased rate of PPE use and societal changes like social distancing. A prospective study of pediatric patients in 7 US cities found that rates of RSV and influenza dramatically decreased during the COVID-19 pandemic.^23^ Although this study did not focus on pediatric HSCT patients, any decrease in viral infections in this patient population could confer survival benefits.

To further investigate rates of nosocomial respiratory viral infections pre- and post-COVID-19 pandemic, and the effects of new respiratory viral pathogen (RVP) positivity on mortality and morbidity in pediatric HSCT patients, we conducted a retrospective, single institution review. Although other studies have analyzed rates of infection pre- and postpandemic, we investigated how all viral patterns changed based on our local infection control practices, and how variables relevant to HSCT outcomes were modulated by nosocomial respiratory viral infections.

## Methods

### Patients

This study was approved by the institutional review board at the University of Chicago. All patients who underwent HSCT, including chimeric antigen receptor T-cell (CAR-T) therapy, between 7/1/2013 and 12/19/2021 at the University of Chicago’s Comer Children’s Hospital were included in the study. There were no excluded patients, including those who received more than 1 HSCT. The pre-COVID-19 precaution era was defined as occurring from 7/1/2013 to 3/17/2020, and the COVID-19 precaution era was defined as occurring from 3/18/2020 to 12/19/2021. This era was further subdivided into early (3/18/2020 to 2/21/2021) and late (2/22/2021 to 12/19/2021), based on the institution’s infection control policies in place during those times. Prior to the pandemic, all HSCT patients had an RVP test on admission and were placed in specialized rooms with pressured double doors, allowing for positive pressure ventilation. Visitor restriction only occurred if a family member was ill, and no pediatric-aged siblings could visit prior to discharge. In comparison, the start of the COVID-19 era was chosen based initiation of visitor restrictions of only 1 parent/guardian at the bedside at a time for pediatric patients, temperature checks on all patients entering the hospital, universal screening of guests for COVID-like symptoms, and stricter social distancing policies of at least 6 feet. Universal masking for all hospital staff, patients, and visitors was required, and staff wore eye protection when interacting with patients. Social distancing, including limiting the number of clinicians entering rooms for rounds or in the physician workrooms, was enacted, and there were times during the pandemic when only 1 physician was allowed to enter a patient’s room, with the remainder of the team participating electronically through a video-based livestream. Additionally, healthcare workers were required to stay home with any respiratory symptoms, even if due to another process, and only return after a negative COVID-19 test and complete resolution of symptoms. If staff tested positive, they were not allowed to return to work until resolution of symptoms or 10 days from their positive test, whichever was longer. When any patient was admitted to the inpatient unit, a SARS-CoV-2 test was required, and the patient was placed on special respiratory precautions (mandatory eye protection, gown, gloves, and N-95 for hospital staff) with no family members able to leave the room, until the test was negative. Finally, all hospital employees were required to attest via a badge swipe that upon entering the hospital, they had no symptoms of a COVID-like illness.

The late COVID-19 era was chosen based on when restrictions first loosened, with adult inpatients able to have visitors and pediatric inpatients allowed to have both parents or 2 guardians at bedside. Further loosening of restrictions came in the following months, including allowing more people to accompany patients to outpatient visits and downgrading of the staff restrictions, including increasing the number of providers or healthcare workers allowed in a room at one time. Universal masking of healthcare workers and visitors continued to be required at our hospital system, as was eye protection when healthcare workers interacted with patients. All healthcare workers had to be vaccinated against SARS-CoV-2 using a 2-dose series unless they had an exemption, with the option to obtain booster shots. During this era, the vaccine was not yet available for pediatric patients under 5 years old outside of a clinical trial, and with Omicron’s ability to cause breakthrough infections, healthcare workers with direct patient contact were required to wear N-95 masks for all patients with suspected SARS-CoV-2 until this variant subsided.

Baseline patient characteristics groups are summarized in Table 1, and patients analyzed per era are detailed in Table 2.

**Table 1.** Patient characteristics for all transplant admissions

Variable	Pre-COVID (n=147)	Post-COVID (n=43)	*P*-value
**Median age (years, range)**	11 (0–34)	7 (1–24)	0.42
**Male (*n*, %)**	74 (50.3%)	27 (62.8%)	0.15
**Transplant type (n)**			0.53
**Allogeneic**	65	15	
**Autologous**	78^a^	27	
**CAR-T**	4	1	
**Allogeneic**			
**ALL/AML/JMML/CML/MDS**	35		
**Lymphoma**	7	0	
**Primary immunodeficiency**	8	0	
**Hemoglobinopathy**	5	3	
**Bone marrow failure/aplastic anemia**	8	1	
**Other**	2	1	
**Autologous**			
**Lymphoma**	10	3	
**Central nervous system tumor**	5	0	
**Neuroblastoma**	52	19	
**Other solid tumor**	11	5	
**CAR-T**			
**ALL**	4	1	

ALL, acute lymphoblastic leukemia; AML, acute myeloid leukemia; CAR-T, chimeric antigen receptor T-cell; CML, chronic myeloid leukemia; JMML, juvenile myelomonocytic leukemia; MDS, myelodysplastic syndrome.

a There were 3 patients who remained inpatient for tandem or triple-tandem hematopoietic stem cell transplantation, whereas others were discharged and then re-admitted for their subsequent transplants.

**Table 2.** Patients per analysis by era

Total number of transplant admissions	Pre-COVID (n=147)	Post-COVID (n=43)
**Total number of stem cell infusions**	150	44
**Incorporated into neutrophil engraftment analysis**	144	43
**Incorporated into platelet engraftment analysis**	128	40

### Data collection

Data was manually collected from electronic medical charts and included date of admission, date of discharge or death, dates of procedures and tests, day of neutrophil engraftment (first day of absolute neutrophil count >500/uL for 3 consecutive days), day of platelet engraftment (platelet count >20 × 10^9^/L for 7 consecutive days in the absence of a platelet transfusion), RVP polymerase chain reaction (PCR) assay results (both on admission and for symptoms to determine if infection was nosocomial), length of stay, transfer to the intensive care unit, oxygen requirements, incidence and grade (I–IV) of acute graft-versus-host disease (GVHD), and overall survival. All inpatient RVP test results, including those of HSCT patients, from the University of Chicago, Comer Children’s Hospital from 7/1/2013 to 12/19/2021 were also obtained to evaluate hospitalwide patterns. Our in-house assay includes adenovirus, coronavirus 229E, HKU1, NL63, and OC43, human metapneumovirus, rhinovirus/enterovirus, influenza A (H1 2009, H1, and H3 subtypes), influenza B, parainfluenza 1–4, RSV, *Bordetella pertussis*, *Chlamydophila pneumoniae*, and *Mycoplasma pneumoniae*. Viral testing was performed via a PCR nucleic acid amplification test, using the BioFire Torch system, obtained from a flocked nasopharyngeal swab. Throughout the study period, the only change in this assay was the addition of SARS-CoV-2 (not included in this analysis).

### Statistical analysis

RVP positivity was calculated by determining the number of patients receiving an HSCT who developed new RVP positivity during that admission, divided by the total number of HSCT that occurred during that era. Differences in the rates of infection in the pre- and COVID-19 eras were compared using an unpaired t-test, as was mean day of neutrophil and platelet engraftment and length of hospitalization stay (LOS). Outcomes, including death within 100, 180, or 365 days from infusion, rates of ICU admission, new oxygen requirement, endotracheal intubation, and incidence of acute GVHD, were performed using a Chi-squared test or a Fisher’s exact test based on the sample size. Incidence of different viral infections was compared using a Fisher’s exact test in analyzing the hospitalwide RVP data.

## Results

### Patient characteristics

The pre-COVID-19 era had 147 HSCT admissions including 65 allogeneic, 78 autologous, and 4 CAR-T, encompassing 133 unique patients (Table 1). The COVID-19 era had 43 HSCT admissions including 15 allogeneic, 27 autologous, and 1 CAR-T, encompassing 31 unique patients. All patients received inpatient transplantation. For those receiving autologous HSCT, there were 3 patients who remained inpatients for tandem or triple-tandem transplants, whereas others were discharged and then re-admitted for subsequent transplantation. Two autologous HSCT patients underwent their first admission in the pre-COVID-19 era and their second in the COVID-19 era. In the pre-COVID-19 era, 3 allogeneic HSCT patients were excluded from neutrophil engraftment analysis, all of whom had severe combined immunodeficiency and failed to engraft. These same 3 patients, in addition to 12 others, died prior to platelet engraftment, and the 4 CAR-T patients were excluded from the pre-COVID-19 platelet engraftment analysis because they never developed a drop in their platelet counts. For allogeneic HSCT patients, 3 patients developed RVP positivity after PMN engraftment, and 3 patient developed RVP positivity after platelet engraftment. For patients undergoing autologous HSCT, 2 patients developed RVP positivity after platelet engraftment. In the COVID-19 analysis, no patients were excluded from neutrophil engraftment analysis, and there were 3 patients excluded from the platelet engraftment analysis. One was excluded because they were transferred to another institution prior to platelet engraftment, one because of no platelet engraftment despite being day +150, and the other was a CAR-T patient who never experienced platelet engraftment due to demise from progressive disease. No patients were censured from the final analyses (Table 2).

### Outcomes with new RVP positivity

**Figure 1.**
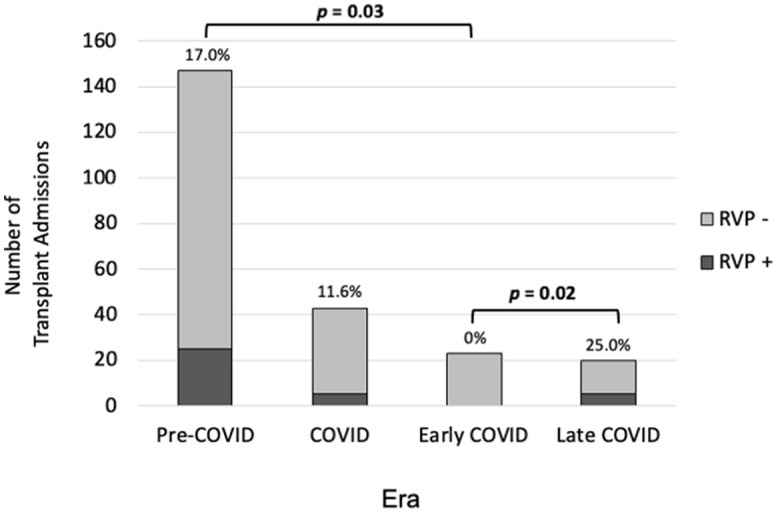
Hospital-acquired respiratory viral pathogen (RVP) positivity in pediatric hematopoietic stem cell transplantation (HSCT) patients by era. The early COVID era demonstrated a complete absence of new RVP positivity, while the pre-COVID and late COVID eras had similar rates of RVP positivity.

The pre-COVID-19 era had a hospital-acquired RVP positivity rate of 20.3% and the total COVID era had a hospital-acquired RVP positivity rate of 11.6%. The measured change was largely due to a significant decrease in RVP positivity during the early COVID era (0%). RVP positivity quickly rebounded in the late COVID era to 25% despite continued, albeit less restrictive, COVID-related hospital infection control policies (Figure 1). This rate of RVP positivity was not significantly different compared to that during the pre-COVID era.

We expected to identify multiple differences in outcome measures in patients who developed new RVP positivity, regardless of the type of transplant or era. Patients were grouped together based on RVP status and not by era, as changes in infection control practices were not expected to influence outcomes once a patient initially developed a nosocomial infection. Unexpectedly, there were no significant differences in any measured outcome following new RVP positivity in autologous HSCT patients compared to those without RVP positivity (Table 3). Only for patients who underwent allogeneic HSCT was the rate of pediatric intensive care unit (PICU) admission, subsequent intubation, and oxygen requirement significant between those who developed RVP positivity and those who did not (Table 4). Nosocomial infections were expected to increase LOS and time to engraftment for patients yet to engraft at time of infection, but these parameters were similar regardless of RVP positivity for both HSCT patient cohorts. Additionally, the rates of acute GVHD were not different between groups. There were 2 allogeneic HSCT patients who required extracorporeal membrane oxygenation with new RVP positivity, but the sample size was too small for any significance to be identified.

**Table 3.** Autologous outcomes based on new RVP +

Variable	New RVP + (n=8)	No new RVP + (n=97)	*P*-value
**Death < 100 days (*n*, %)**	1 (12.5%)	2 (2.1%)	0.09
**Death < 180 days (*n*, %)**	1 (12.5%)	6 (6.2%)	0.49
**Death < 365 days (*n*, %)**	1 (12.5%)	9 (9.3%)	0.77
**PICU admission (*n*, %)**	2 (25.0%)	22 (22.7%)	0.88
**Oxygen requirement *(n*, %)**	2 (25.0%)	26 (26.8%)	0.91
**Intubation (*n*, %)**	2 (25.0%)	7 (7.2%)	0.08
**LOS (mean days)**	37.6	34.1	0.47
**Neutrophil engraftment (mean day)**	10.6	11.1	0.52
**Platelet engraftment (mean day)**	28.0	20.9^a^	0.14

LOS, length of stay; PICU, pediatric intensive care unit.

a Includes 2 patients with RVP positivity that occurred post-engraftment.

**Table 4.** Allogeneic outcomes based on new RVP +

Variable	New RVP + (n=21)	No new RVP + (n=59)	P-value
**Death < 100 days (*n*, %)**	6 (28.6%)	7 (11.9%)	0.07
**Death < 180 days (*n*, %)**	7 (33.3%)	9 (15.3%)	0.08
**Death < 365 days (*n*, %)**	7 (33.3%)	13 (22.0%)	0.30
**PICU admission (*n*, %)**	14 (66.7%)	19 (32.2%)	0.01
**Oxygen requirement (*n*, %)**	14 (66.7%)	24 (40.7%)	0.04
**Intubation (*n*, %)**	12 (57.1%)	11 (18.6%)	< 0.01
**LOS (mean days)**	75.9	60.0	0.17
**Neutrophil engraftment (mean day)**	21.9	20.7^a^	0.58
**Platelet engraftment (mean day)**	34.3	31.7^b^	0.72
**GvHD grades I/II**	5 (23.8%)	8 (13.6%)	0.27
**GvHD grades III/IV**	0 (0%)	3 (5.1%)	0.56

GvHD, graft-versus-host disease; LOS, length of stay; PICU, pediatric intensive care unit.

a Includes 3 patients with RVP positivity that occurred post-engraftment.

b Includes 1 patient with RVP positivity that occurred post-engraftment.

### Hospital respiratory pathogen data

**Figure 2.**
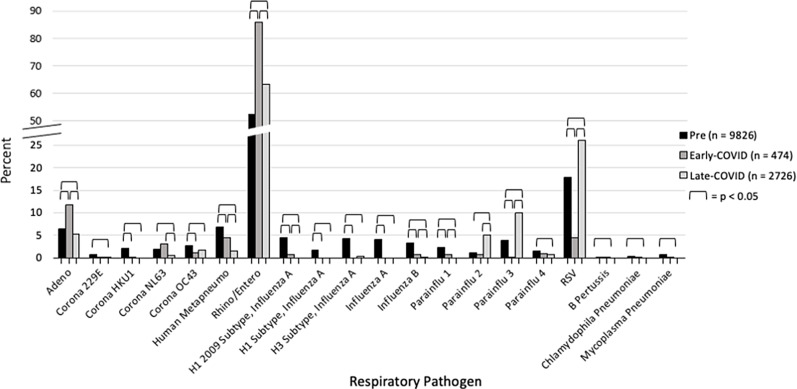
Hospital-wide respiratory viral pathogen (RVP) data by era.

RVP testing from all newly admitted symptomatic patients was collected at our hospital from the pre- and COVID-19 eras to compare incidence rates of different viral infections and the changes brought about from the different PPE usage rates of the pandemic. A total of 10,244, 501, and 2,885 RVP tests were sent during the pre-COVID-19, early COVID-19, and late COVID-19 era, respectively. There were similar rates of positivity throughout the different eras. There was 95.9% positivity (9,826 positive tests) during the pre-COVID-19 era, 94.6% (474 positive tests) during the early COVID-19 era, and 94.5% (2,726 positive tests) in the COVID-19 eras. However, there were significantly different positivity rates for individual pathogens during each era (Figure 2).

Rhinovirus/enterovirus was the most acquired virus during all 3 eras at 52.54% pre-COVID, 85.84% early, and 63.35% late COVID era (Figure 2). Adenovirus accounted for the second largest number of infections during the early COVID era (13% vs 6.4% pre-COVID and 5.3% late COVID). The dominance of these viruses during the early COVID era was in large part the result of near complete absence of other normally detected pathogens including influenza and parainfluenza viruses. Interestingly, the rates of the 3 dominant viruses (rhinovirus/enterovirus, RSV, and adenovirus) between the pre-COVID and late COVID eras were similar (Figure 2).

**Figure 3.**
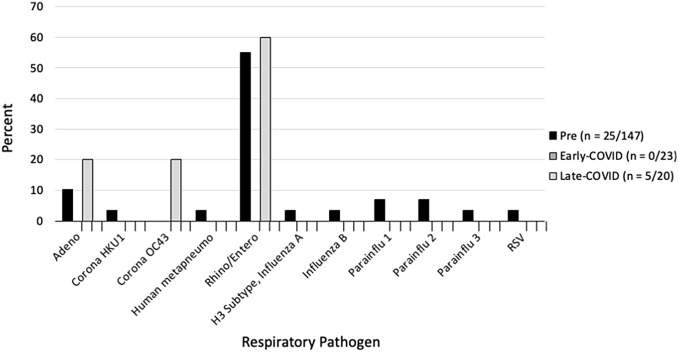
Respiratory pathogens identified in hematopoietic stem cell transplantation (HSCT) patients by era.

The normalization of RVP positivity patterns in the general pediatric population between pre-COVID and late COVID eras (Figure 2) was also reflected in nosocomial infections in HSCT patients (Figure 3). Although there was a greater diversity of viruses represented during the pre-COVID era, infections in HSCT were equally dominated by rhinovirus/enterovirus (55.2%, 60.0%) and adenovirus (10.3%, 20.0%) during the pre-COVID and late COVID eras, respectively.

## Discussion

In this retrospective review of pediatric HSCT admissions that occurred during the pre- and post-COVID-19 pandemic, we identified significant differences in RVP positivity due to changes made in hospital and social practices during early stages of the pandemic, and for patients with new RVP positivity during their admission, changes in patient outcomes. After the advent of the pandemic, our rates of new RVP positivity dropped to zero, compared to the 20.3% rate prior to the pandemic, which then returned to prepandemic levels with the slightest loosening of restrictions. This early COVID era was short, lasting less than a year, but throughout this era, we found that constant attention to strict infection control practices dramatically reduced the risk of nosocomial infections, even during the winter months when these infections are generally more common. New RVP positivity was associated with increased incidence of PICU admission, intubation, and oxygenation in allogeneic HSCT patients, but preventing these infections required a concerted effort by society and hospital infection control measures to maintain. Unless a similar pandemic state occurs in the future, it is unlikely that society and hospitals will revert to the strictest measures in place in the early part of the pandemic, and that hospital-acquired RVP positivity will continue to result in complications for HSCT patients.

For our hospital, the prevalence of specific viral infections was altered by the pandemic, with most of the tested respiratory pathogens undergoing significant changes. This was true in tests isolated from our pediatric HSCT patients, in that although adenovirus and rhinovirus/enterovirus remained common etiologies for a hospital-acquired infection, similar to prior literature,^24^ after the start of the pandemic, there were no HSCT patients who acquired influenza, parainfluenza, or RSV. Although adenovirus can be treated with cidofovir or brincidofovir, these medications are not without side effects, and most other respiratory infections lack a directed therapy.

Our facility does not have a separate HSCT unit, with the patients undergoing HSCT residing on a general pediatrics floor, although they board in specialized rooms with double doors which allow for positive pressure ventilation within the room and negative pressure in the anteroom. All patients continue to be placed on contact and droplet isolation regardless of symptoms, and the number of visitors is limited to 2 caregivers. Despite this, risks remain for nosocomial viral infections, and as shown, any loosening of restrictions, such as less stringent universal screening and changes in societal attitudes toward SARS-CoV-2, resulted in rates of new hospital-acquired RVP positivity return to prepandemic levels. Other studies have found that visitor restrictions did not influence nosocomial viral respiratory infections, arguing that it was other myriad infection control measures that were responsible for the decrease in respiratory infections during the pandemic.^25^ Universal masking of all healthcare workers has been found to correlate with decreased hospital-acquired respiratory viral infections during the pandemic,^26^ which could be a cost-effective method to reduce morbidity and mortality in HSCT patients.

As the pandemic moves further and further into the past, it is important to consider what gains were made during the pandemic for this vulnerable patient population and consider continuing some of these restrictions, even universal masking of healthcare providers, to optimize patient outcomes.

## References

[1] Novelli, G , Biancolella, M , Mehrian-Shai, R , et al. COVID-19 update: the first 6 months of the pandemic. Hum Genomics 2020;14:48. doi:10.1186/s40246-020-00298-w. 33357238 PMC7757844

[2] Duggal M , Dahiya N , Kankaria A , Chaudhary M , Bachani D. Restructuring the healthcare system to protect healthcare personnel amidst the COVID-19 pandemic. Front Public Health 2020;8:588203. doi:10.3389/fpubh.2020.588203. 33363085 PMC7759646

[3] WHO Director-General’s opening remarks at the media briefing on COVID-19 - 11 March 2020. https://www.who.int/director-general/speeches/detail/who-director-general-s-opening-remarks-at-the-media-briefing-on-covid-19---11-march-2020. Accessed November 17, 2021.

[4] Considerations for public health and social measures in the workplace in the context of COVID-19. https://www.who.int/publications-detail-redirect/WHO-2019-nCoV-Adjusting_PH_measures-Workplaces-2020.1. Accessed November 17, 2021.

[5] CDC. COVID-19 ARCHIVED WEBPAGE. Centers for disease control and prevention, February 11, 2020. https://www.cdc.gov/coronavirus/2019-nCoV/index.html. Accessed November 17, 2021.

[6] Waghmare A , Abidi MZ , Boeckh M , et al. Guidelines for COVID-19 management in hematopoietic cell transplantation and cellular therapy recipients. Biol Blood Marrow Transplant 2020;26:1983–1994. doi:10.1016/j.bbmt.2020.07.027. 32736007 PMC7386267

[7] Jabarpour M , Dehghan M , Afsharipour G , et al. The impact of COVID-19 outbreak on nosocomial infection rate: a case of Iran, *Can J Infect Dis Med Microbiol* 2021;6650920:1–6. 10.1155/2021/6650920 PMC7905999 33680220

[8] De la Puerta R , Montoro J , Aznar C , et al. Common seasonal respiratory virus infections in allogeneic stem cell transplant recipients during the SARS-COV-2 pandemic. Bone Marrow Transplant 2021;56:2212–2220. doi:10.1038/s41409-021-01319-5. 33947980 PMC8093913

[9] Raad, MD I , Abbas, MD J , Whimbey, MD E. Infection control of nosocomial respiratory viral disease in the immunocompromised host. Am J Med 1997;102:48–52. doi:10.1016/S0002-9343(97)00011-9. 10868143

[10] Sokol KA , De la Vega-Diaz I , Edmondson-Martin K , et al. Masks for prevention of respiratory viruses on the BMT unit: results of a quality initiative. Transpl Infect Dis 2016;18:965–967. doi:10.1111/tid.12608. 27632416

[11] Sung AD , Sung JAM , Thomas S , et al. Universal mask usage for reduction of respiratory viral infections after stem cell transplant: a prospective trial. Clin Infect Dis 2016;63:999–1006. doi:10.1093/cid/ciw451. 27481873 PMC5036914

[12] Sharma A , Bhatt NS , St Martin A , et al. Clinical characteristics and outcomes of COVID-19 in haematopoietic stem-cell transplantation recipients: an observational cohort study. Lancet Haematol 2021;8:e185–e193. doi:10.1016/S2352-3026(20)30429-4. 33482113 PMC7816949

[13] Vicent MG , Martinez AP , Trabazo del Castillo M , et al. COVID-19 in pediatric hematopoietic stem cell transplantation: the experience of Spanish Group of Transplant (GETMON/GETH). Pediatr Blood Cancer 2020;67(9):e28514.1–2. doi: 10.1002/pbc.28514. PMC7361142 32573924

[14] Chow EJ , Mermel LA. Hospital-acquired respiratory viral infections: incidence, morbidity, and mortality in pediatric and adult patients. Open Forum Infect Dis 2017;4:ofx006. doi:10.1093/ofid/ofx006. 28480279 PMC5414085

[15] Piñana JL , Martínez-López C , Chorão P , et al. Characterizing respiratory virus infections during the peri-engraftment period of allogeneic hematopoietic cell transplant. Transpl Infect Dis 2025;Sep 11:e70101. doi:10.1111/tid.70101. 40932461

[16] Pochon C , Voigt S. Respiratory virus infections in hematopoietic cell transplant recipients. Front Microbiol 2019;9:3294. doi:10.3389/fmicb.2018.03294. 30687278 PMC6333648

[17] Fisher BT , Danziger-Isakov L , Sweet LR , et al. A multicenter consortium to define the epidemiology and outcomes of inpatient respiratory viral infections in pediatric hematopoietic stem cell transplant recipients. J Pediatr Infect Dis Soc 2018;7:275–282. doi:10.1093/jpids/pix051. PMC7107490 29106589

[18] Ariza-Heredia EJ , Chemaly RF. Update on infection control practices in cancer hospitals. Cancer J Clin 2018;68:340–355. doi:10.3322/caac.21462. PMC7162018 29985544

[19] Khawaja F , Chemaly RF. Respiratory syncytial virus in hematopoietic cell transplant recipients and patients with hematologic malignancies. Haematologica 2019;104:1322–1331. doi:10.3324/haematol.2018.215152. 31221784 PMC6601091

[20] Faizan M , Caniza MA , Anwar S , et al. Infection prevention and control measures at the children hospital Lahore: a my child matters collaborative project. JCO Glob Oncol 2020;1540–1545. doi:10.1200/GO.20.00403. 33064627 PMC7605375

[21] Ward S , Lindsley A , Courter J , Assa’ad A. Clinical testing for COVID-19. J Allergy Clin Immunol 2020;146:23–34. doi:10.1016/j.jaci.2020.05.012. 32445839 PMC7237919

[22] Olsen SJ , Winn AK , Budd AP , et al. Changes in influenza and other respiratory virus activity during the COVID-19 pandemic—United States, 2020–2021. Am J Transplant 2021;21:3481–3486. doi:10.1111/ajt.16049. 34624182 PMC8653380

[23] Haddadin Z , Schuster JE , Spieker AJ , et al. Acute respiratory illnesses in children in the SARS-CoV-2 pandemic: prospective multicenter study. Pediatrics 2021;148:e2021051462. doi:10.1542/peds.2021-051462. 33986150 PMC8338906

[24] Renaud C , Campbell AP. Changing epidemiology of respiratory viral infections in hematopoietic cell transplant recipients and solid organ transplant recipients. Curr Opin Infect Dis 2011;24:333–343. doi:10.1097/QCO.0b013e3283480440. 21666460 PMC3210111

[25] Wee LE , Conceicao EP , Sim JXY , Aung MK , Venkatachalam I. The impact of visitor restrictions on health care-associated respiratory viral infections during the COVID-19 pandemic: experience of a tertiary hospital in Singapore. Am J Infect Control 2021;49:134–135. doi:10.1016/j.ajic.2020.11.006. 33186677 PMC7654321

[26] Wong SC , Lam GKM , AuYeung CHY , et al. Absence of nosocomial influenza and respiratory syncytial virus infection in the coronavirus disease 2019 (COVID-19) era: implication of universal masking in hospitals. Infect Control Hosp Epidemiol: 1–4. doi:10.1017/ice.2020.425 PMC7468684 32799965

